# RNAi in the Rhizarian Phytopathogen *Plasmodiophora brassicae*: The Causal Agent of Clubroot Disease in Cruciferous Crops

**DOI:** 10.1002/advs.202524028

**Published:** 2026-09-06

**Authors:** Xiong Zhang, Yupo Wu, Jiachang Hou, Lixia Gao, Longqi Pan, Cuicui Shen, Chuanji Zhao, Feng Gao, Yi Zhang, Li Ren, Xiaoping Fang, Xiaoling Dun, Shengyi Liu, Lijiang Liu

**Affiliations:** ^1^ Oil Crops Research Institute of the Chinese Academy of Agricultural Sciences/The Key Laboratory of Biology and Genetic Improvement of Oil Crops The Ministry of Agriculture and Rural Affairs Wuhan China; ^2^ Yichang Academy of Agricultural Sciences Yichang Hubei China

**Keywords:** clubroot disease, cruciferous crops, *Plasmodiophora brassicae*, Rhizaria, RNA interference (RNAi)

## Abstract

Although RNA interference (RNAi) is widespread and functionally important across eukaryotes, RNAi pathways are diverse or even lost in some lineages. Rhizaria represents a major and distinct eukaryotic supergroup that includes *Plasmodiophora brassicae* (*Pb*), the causal agent of cruciferous clubroot disease, yet RNAi in this lineage remains poorly understood. Here, we characterized an unusual RNAi pathway in *Pb*. Small RNA sequencing across five representative *Pb* life stages revealed abundant siRNAs and miRNAs characterized by a predominant 21‐nt length, phased genomic distribution, 2‐nt 3′ overhangs, and a strong 5′‐cytidine bias. Three *Pb* miRNAs were further validated by northern blotting and stem‐loop RT–qPCR. Genome analysis identified two canonical AGO homologs, PbAGO1 and PbAGO2, but no Dicer homologs, except for an RNase III‐containing Drosha‐like protein, PbDRL. Functional analyses showed that PbAGO1 and PbAGO2 mediate gene silencing, whereas PbDRL is required for sRNA biogenesis. Further, the cell wall component chitin was identified from *Pb* zoosporangia during the early infection and RNAi interfering with its biosynthesis in transgenic plants of Arabidopsis and *Brassica napus* blocked *Pb* early infection and conferred broad‐spectrum resistance. Our study uncovers an unusual RNAi pathway in Rhizaria and provides a promising strategy to control cruciferous clubroot disease.

## Graphical abstract

This study uncovers an unusual RNAi pathway in the rhizarian pathogen *Plasmodiophora brassicae*. In the absence of Dicer, a Drosha‐like RNase III protein supports the biogenesis of predominant 21‐nt small RNAs, and two Argonaute proteins mediate small RNA‐guided silencing. These findings provide new insights into RNAi in Rhizaria and expand current understanding of RNAi diversity across eukaryotes.

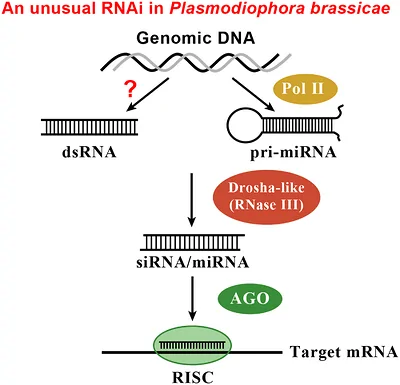

## 1 Introduction

RNA interference (RNAi), triggered by small RNAs (sRNAs; ∼20–30 nucleotides, nt), is a conserved mechanism across diverse eukaryotic lineages. It represses gene expression either transcriptionally, through DNA methylation, or post‐transcriptionally, via mRNA cleavage and/or translational inhibition, thereby regulating a wide range of biological processes [1]. In the simplest model of RNAi pathway, DNA/RNA‐dependent RNA polymerase (DdRP/RdRP) generates long double‐stranded RNAs (dsRNAs), which are subsequently cleaved into sRNAs by the RNase III endonuclease Dicer, or by Dicer together with Drosha in animals. The effector protein Argonaute (AGO) then binds these sRNAs and uses them to guide sequence‐specific cleavage of target transcripts, thereby inducing gene silencing [2, 3]. Nevertheless, RNAi pathways have evolved considerable genetic diversity among eukaryotic species. For example, in plants, fungi, and oomycetes, the core components for sRNA biogenesis typically consist of DdRP, RdRP, and Dicer [3, 4, 5], whereas in animals, DdRP, Dicer and Drosha are the principal components involved in the generation of sRNAs [6]. Consequently, in plants, fungi, and oomycetes, two major classes of sRNAs‐small interfering RNAs (siRNAs) and microRNAs (miRNAs)‐are generated, whereas animals predominantly produce miRNAs [3, 4, 5, 6]. In addition, Dicer‐independent sRNA biogenesis pathways have also been reported in certain eukaryotes, such as budding yeast, mice, and Arabidopsis [7, 8, 9]. Moreover, the number of AGO family members varies greatly among species, ranging from one in many organisms to 10 in Arabidopsis [10], and as many as 27 members in nematodes [11], where they are functionally differentiated and play distinct roles in diverse biological processes. Remarkably, RNAi pathways have even been lost in some eukaryotic species, including *Saccharomyces cerevisiae*, *Plasmodium falciparum*, and *Ustilago maydis* [12].

Rhizaria is a major eukaryotic supergroup of unicellular and predominantly heterotrophic organisms lacking clear morphological or structural synapomorphies and, together with Stramenopila and Alveolata, may account for nearly half of all eukaryotic diversity [13, 14, 15]. Rhizaria includes ecologically important marine and terrestrial protists that occupy the base of food webs and form a major link in the transfer of energy to higher‐order consumers [13]. Most rhizarian species are free‐living, while only a small number are parasitic and primarily infect crops or invertebrates. To date, no infamous human pathogens have been identified in this group, an unusual phenomenon among eukaryotic supergroups [13, 14]. In addition, most rhizarian species are difficult to cultivate. Consequently, even in the era of metagenomics and high‐throughput sequencing, Rhizaria remains understudied, and available reference genomes are still limited. At present, RNAi in Rhizaria remains largely unexplored.

Within Rhizaria, *Plasmodiophora brassicae *(*Pb*) is a representative member that can infect plants and causes notorious clubroot disease to cruciferous plants [16, 17]. Currently, clubroot disease has been reported in more than 80 countries and has resulted in annual yield losses of 10%–15% worldwide [18, 19]. Unlike most phytopathogens which are extracellular, *Pb* has an intracellular biotrophic lifestyle [17, 20], indicating a unique and intimate interaction with host plants. The life cycle of *Pb* is complex and recently clarified, generally including the resting spores in soil, the primary infection in root epidermis tissue, and the secondary infection in root cortex tissue [21]. However, the molecular mechanisms underlying *Pb* biology and its interactions with host plants remain poorly understood, largely due to the lack of genetic manipulation tools. RNAi has been widely used to study gene function in many species, particularly in obligate biotrophic fungi and oomycetes [22]. However, whether RNAi is present and functional in *Pb* remains unclear.

Utilization of clubroot resistance genes in breeding is a major strategy for controlling clubroot disease in cruciferous crops. Unfortunately, specialized pathotypes of *Pb* frequently overcame the resistance of resistant cruciferous varieties [23, 24, 25]. Moreover, accumulating evidence supports that host resistance, even at the immune level, can block secondary infection but not primary infection [21, 26, 27], thereby accelerating resistance breakdown and disease spread. Broad‐spectrum resistance genes are promising for durable control of clubroot disease but are still very limited, although two ones have been reported [26, 27]. Therefore, it is important to identify new targets and develop new management strategies for clubroot disease control. Owing to the high efficiency, target specificity, and capacity for simultaneous multi‐targeting, RNAi technology has recently emerged as a promising approach for controlling a wide range of agricultural diseases and represents a new generation of eco‐friendly technologies [28, 29]. However, promising targets for the control of clubroot disease have rarely been reported.

In this study, we characterized an unusual RNAi landscape in *Pb*, expanding our understanding of RNAi diversity across eukaryotes. In addition, we identified chitin, a cell wall component of zoosporangia formed during the early stages of *Pb* infection, as an RNAi target, and created engineered transgenic plants with broad‐spectrum resistance to different *Pb* pathotypes.

## 2 Results

### 2.1 Multi‐Omics Profiling and Genome Assembly of a New *Pb* Isolate

**FIGURE 1:**
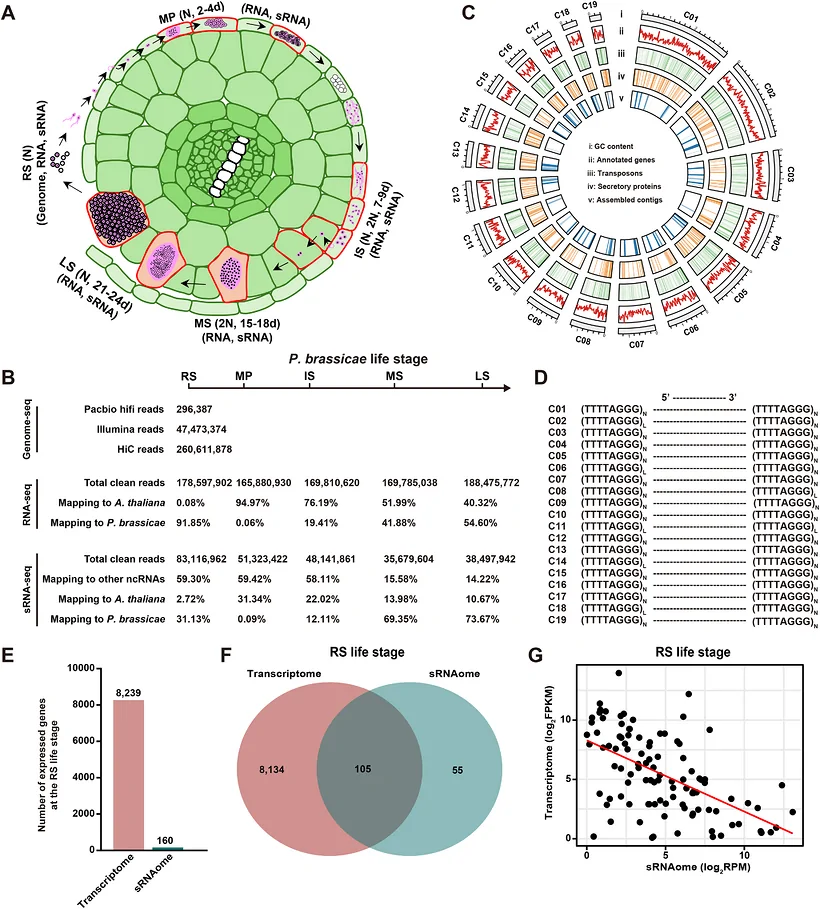
Multi‐omics analysis of the isolate *PbJS* across five representative life stages. (A) Schematic illustration of the five representative life stages in the *Pb* life cycle used for genome, small RNA, and transcriptome sequencing. The *Pb* life cycle was adapted from our previous study [17]. RS, resting spores (haploid, N); MP, the middle primary infection (2–4 dpi), including the haploid life forms multinucleate primary plasmodia and zoosporangia in the root epidermal cells; IS, initiation of the secondary infection (7–9 dpi), including the haploid life forms secondary zoospores, zygotes and cysts, and the early diploid (2N) life forms of the secondary infection‐the uninucleate and binucleate secondary plasmodia; MS, middle secondary infection (15–18 dpi), including the diploid multinucleate secondary plasmodia that cause extensive expansion of infected cortical cells; LS, late secondary infection (21–24 dpi), including the diploid massive multinucleate secondary plasmodia, the haploid resting sporangial plasmodia and immature next‐generation haploid resting spores. (B) Overview of the sequencing data obtained from five *Pb* life stages. (C) Circos plot showing the genomic features of the PbJS isolate. The outermost circle represents the 19 assembled chromosomes (i), and the inner circles represent the distributions of GC content (ii), annotated genes (iii), transposons (iv), and predicted secreted proteins (v). (D) Conserved repeat motifs at both ends of each assembled PbJS chromosome. N denotes multiple repeats of the TTTTAGGG motif, whereas L denotes multiple repeats of the TTTTAGGG‐like motif. (E) Number of expressed *Pb* genes at the RS stage identified based on reads generated by transcriptome sequencing and sRNAome sequencing, respectively. (F) Venn diagram of expressed *Pb* genes at the RS stage identified by transcriptome sequencing‐ and sRNAome‐derived reads. (G) Correlation analysis of the expression levels of *Pb* genes identified by both transcriptome sequencing and sRNAome sequencing. Gene expression levels are shown as log2(FPKM) values for transcriptome sequencing and log2(RPM) values for sRNAome sequencing.

The isolate *Plasmodiophora brassicae Jishou* (*PbJS*) was collected in 2022 from diseased *Brassica napus* plants in Jishou County, Hunan Province, and was classified as the pathotype ECD16/23/31, which can overcome the resistance of the *B. napus* cultivar Huashuang 5R [25]. To investigate the RNAi system in *Pb*, this isolate was subjected to multi‐omics profiling including de novo genome sequencing, small RNA sequencing, and transcriptome sequencing. Based on the life cycle recently clarified [17], five representative life stages were selected (Figure 1A): the haploid resting spore stage (RS), the long‐surviving form in soil; the middle primary infection stage (MP, 2–4 days post inoculation, dpi), including the haploid life forms multinucleate primary plasmodia and zoosporangia in the root epidermis; the initiation of secondary infection stage (IS, 7–9 dpi), including the haploid life forms secondary zoospores, zygotes, and cysts, as well as the early diploid life form of the secondary infection‐the uninucleate secondary plasmodia; the middle secondary infection stage (MS, 15–18 dpi), composed of the diploid multinucleate secondary plasmodia that cause massive expansion of infected cortical cells; and the late secondary infection stage (LS, 21–24 dpi), encompassing diploid massive multinucleate secondary plasmodia, haploid resting sporangial plasmodia, and immature next‐generation haploid resting spores. Mature resting spores were purified from diseased root tissues and used for the isolation of genomic DNA, total RNA, and sRNA for deep sequencing, respectively. Root tissues containing the other four representative *Pb* life stages, which could not be isolated separately, were used for transcriptome and sRNAome sequencing.

The genome, transcriptome, and sRNAome sequencing data are summarized in Figure 1B and Tables S1–S5. By integrating the genome and transcriptome sequencing data, we generated a telomere‐to‐telomere genome assembly for *PbJS* with a total size of 25.22 Mb, comprising 19 supercontigs, an N50 of 1.45 Mb, 91.76% genome completeness, and 10 120 annotated genes (Figure 1C,D and Figure S1). After removing low‐quality reads, 3′ and 5′ adapters, and reads shorter than 17 nucleotides (nt), sRNAome sequencing yielded a substantial number of high‐quality sRNAs from root tissues, ranging from 35 679 604 reads in MS root tissue to 83 116 962 reads at the RS stage. In total, 84 885 718 clean reads from the five life stages were identified originally from the *PbJS* genome, excluding genomic regions corresponding to tRNAs or rRNAs (Figure 1B and Figure S2). Comparison of the clean sRNAome reads with transcriptome sequencing data indicated that only a very small proportion of the sRNAome reads represented degradation products derived from highly expressed mRNAs (Figure 1E–G and Figure S3A–D). Collectively, these results provide a high‐quality reference genome for a new *Pb* isolate and identify a substantial set of high‐quality sRNAs.

### 2.2 Identification and Validation of *Pb* siRNAs and miRNAs

**FIGURE 2:**
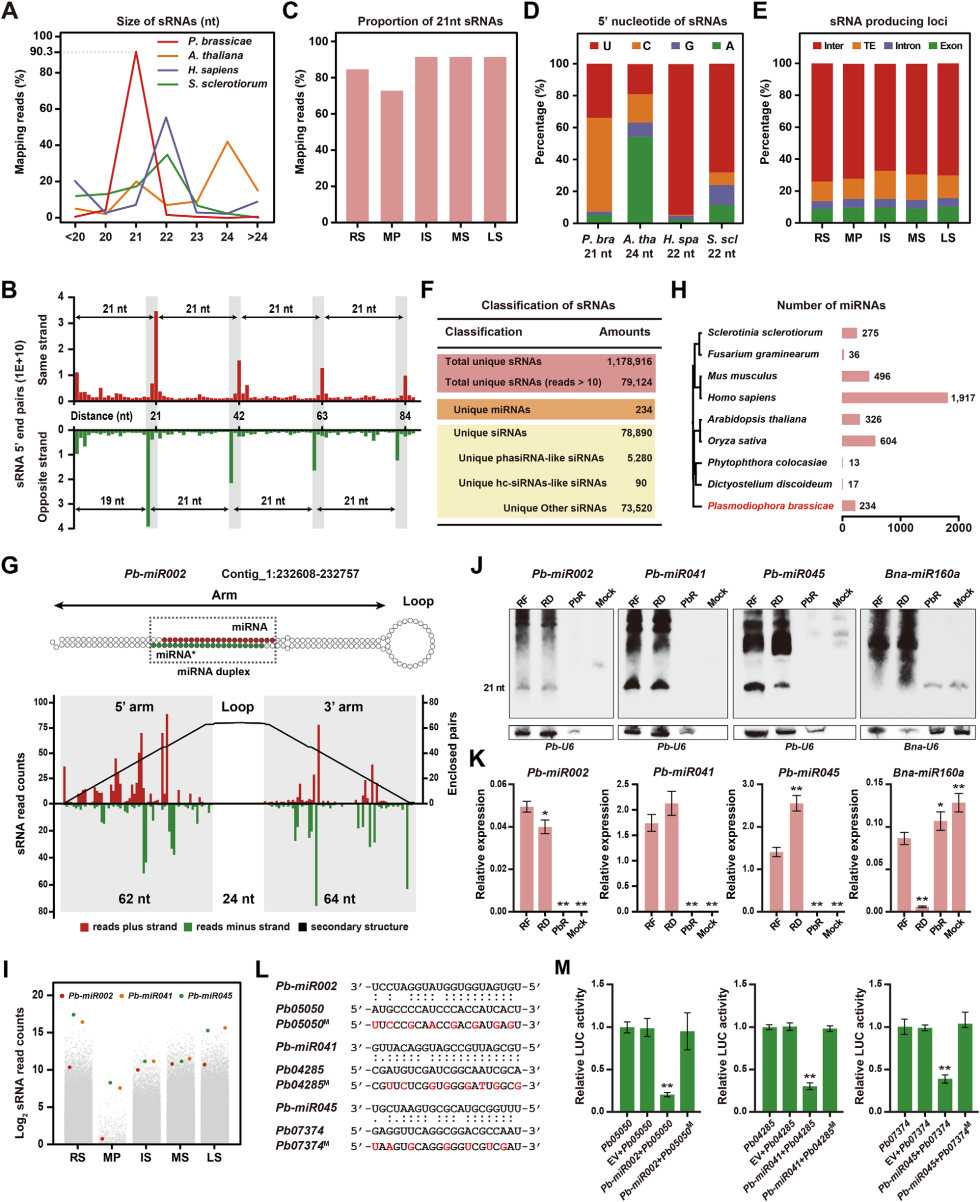
Characterization and validation of *Pb* sRNAs. (A) Size‐distribution profiles of sRNAs in *Pb* and three representative species from plants, animals, and fungi: *Arabidopsis thaliana*, *Homo sapiens*, and *Sclerotinia sclerotiorum*, respectively. (B) Distribution of genomic intervals separating the 5′ termini of sequenced 21‐nt RNAs in *Pb*. The upper panel shows sRNA pairs mapped to the same DNA strand, whereas the lower panel shows sRNA pairs mapped to opposite DNA strands. The frequency of each interval is plotted for all pairs of reads located within 100 nt of each other. (C) Proportion of 21‐nt sRNAs across the five *Pb* life stages. (D) Analysis of 5′‐terminal nucleotide bias in the predominant sRNAs of *Pb*, *A. thaliana*, *H. sapiens*, and *S. sclerotiorum*. (E) Genome‐wide identification of sRNA‐producing loci in the *PbJS* genome. Inter, intergenic regions; TE, transposable elements. (F) Classification of *Pb* sRNAs. (G) A palindromic genomic region that generates miRNAs in *Pb*. The 21‐nt sRNAs are mapped to the genome, and read counts (normalized to the number of genomic matches) are plotted for the plus and minus strands. The predicted secondary structure is shown as a mountain plot. (H) Numbers of miRNAs in *Pb* and eight other representative species from plants, animals, oomycetes, and fungi. miRNA data for *Mus musculus*, *Homo sapiens*, *A. thaliana*, and *Oryza sativa* were obtained from miRBase (https://www.mirbase.org/), whereas data for *S. sclerotiorum*, *Fusarium graminearum*, *P. colocasiae*, and *Dictyostelium discoideum* were obtained as previously described [4, 32, 34, 35]. The species tree was generated using the TimeTree5 web server (http://www.timetree.org/) and visualized with MEGA X software. (I) Read counts of *Pb‐miR002*, *Pb‐miR041*, and *Pb‐miR045* in the deeply sequenced sRNA pools from the five *Pb* life stages. (J) Northern blot analysis of *Pb* miRNAs *Pb‐miR002*, *Pb‐miR041*, and Pb‐miR045, together with the *B. napus* miRNA *Bna‐miR160a*, in fresh clubbed roots of *B. napus* (RF), dry clubbed roots of *B. napus* (DF), *B. napus* roots infected with *Pb* at 23 dpi (PbR), and healthy *B. napus* roots (Mock). *Pb* U6 snRNA was used as the internal control for quantification of *Pb* miRNAs, whereas *B. napus* U6 snRNA was used as the internal control for quantification of the *B. napus* miRNA. (K) Stem‐loop RT‐qPCR validation and quantification of *Pb* miRNAs and *Bna‐miR160a*. (L) Predicted sRNA target sites and their mutated variants (mutations shown in red). (M) Dual‐luciferase reporter assays of sRNA‐mediated cleavage of target transcripts in *Arabidopsis* protoplasts. Values were normalized to Renilla luciferase activity. In (K and M), data are presented as mean ± SD from three biological replicates. Statistical significance was determined by two‐tailed Student's *t*‐test, *p < 0.05, **p < 0.01.

Regulatory small RNAs (sRNAs) often display a characteristic length bias in different species, for example, 24‐ and 21‐nt sRNAs in *A. thaliana* [30], 22‐nt sRNAs in humans [31], and the phytopathogenic fungus *Sclerotinia sclerotiorum* (Figure 2A) [32]. Among all the sequenced sRNAs mapped to *PbJS* genome, 90.3% *Pb* sRNAs are 21 nt in length (Figure 2A and Figure S4A), distinct from those in plants, human, and fungi. Moreover, statistical analysis of the intervals between mapped genomic ends of all sequenced *Pb* sRNAs revealed a clear 21‐nt phasing pattern, whereas pairs derived from opposite strands showed the same phasing interval but with a 2‐nt register offset relative to same‐strand pairs (Figure 2B). This pattern is consistent with the action of classical endonucleases, which have been reported to sequentially cleave double‐stranded RNA precursors while simultaneously generating the 3′ end of one sRNA and the 5′ end of the next [7]. The proportion of 21‐nt sRNAs increased from 82.5% at the RS stage to 92.3% at the IS, MS and LS life stages (Figure 2C). Interestingly, most *Pb* sRNAs began with cytidine (C) at the 5′ end, in contrast to the preference for adenosine (A) in *A. thaliana* and uridine (U) in humans and *S. sclerotiorum* (Figure 2D and Figure S4B). Genomic loci producing sRNAs were identified and were found to originate mainly from intergenic regions, followed by transposable elements, exons, and introns (Figure 2E and Figure S4C). Through integrated analysis of genomic origin, precursor structure, and read abundance, we annotated 234 miRNAs and 78 890 siRNAs from the sequenced *Pb* sRNAs (Figure 2F,G). Among these 78 890 siRNAs, 5280 putative phasiRNA‐like siRNAs and 90 putative hc‐siRNA‐like siRNAs were further identified with sRNAminer (Figure 2F and Tables S6 and S7) [33]. Detailed information on all 234 *Pb* miRNA species and their predicted target genes is provided in Tables S8 and S9. Comparative genomic analysis showed that the number of annotated miRNAs varies greatly among species, and that *Pb* contains substantially more miRNAs than its close relatives *Phytophthora colocasiae* (13) and *Dictyostelium discoideum* (17) (Figure 2H).

Among the 234 identified *Pb* miRNA species, three miRNA species *Pb‐miR002*, *Pb‐miR041*, and *Pb‐miR045* were highly expressed across all five *Pb* life stages (Figure 2I) and were therefore selected for northern blot analysis. Total RNA was isolated from resting spores purified from fresh and dried clubbed root tissues, fresh clubbed root tissues, and healthy root tissues. Northern blot analysis detected strong 21‐nt signals of all three *Pb* miRNAs from the total RNA of resting spores, but not in healthy root tissues (Figure 2J). In contrast, the host plant miRNA *miR160a*, a mature 22‐nt miRNA identified previously with abundant accumulation in the root tissues of *B. napus* [36], was detected with a 22‐nt signal from the total RNA samples of both diseased and healthy root tissues but not in *Pb* resting spores (Figure 2J). Unexpectedly, *Pb‐miR002*, *Pb‐miR041*, and *Pb‐miR045* were not detected in the total RNAs from fresh clubbed root tissues. This was likely due to insufficient accumulation of *Pb* biomass in these tissues, as indicated by the weak fluorescence signal of *Pb* U6 (Figure 2J), a housekeeping gene constitutively expressed across all five *Pb* life stages. We further confirmed the presence of these three *Pb* miRNAs in isolated resting spores by reverse transcription‐quantitative PCR (RT‐qPCR), whereas they remained undetectable in clubbed root tissues (Figure 2K). We next investigated whether *Pb* miRNAs could mediate silencing of their target transcripts using dual‐luciferase transient expression assays in *Arabidopsis* protoplasts. As expected, all three selected *Pb* miRNAs induced silencing of their target genes, as indicated by markedly reduced LUC activity, whereas introduction of mutations into the target sites completely abolished their silencing activity (Figure 2L,M). Taken together, these results demonstrate that *Pb* produces functional sRNAs with unique molecular characteristics.

### 2.3 Identification and Functional Validation of *Pb* AGO Proteins

**FIGURE 3:**
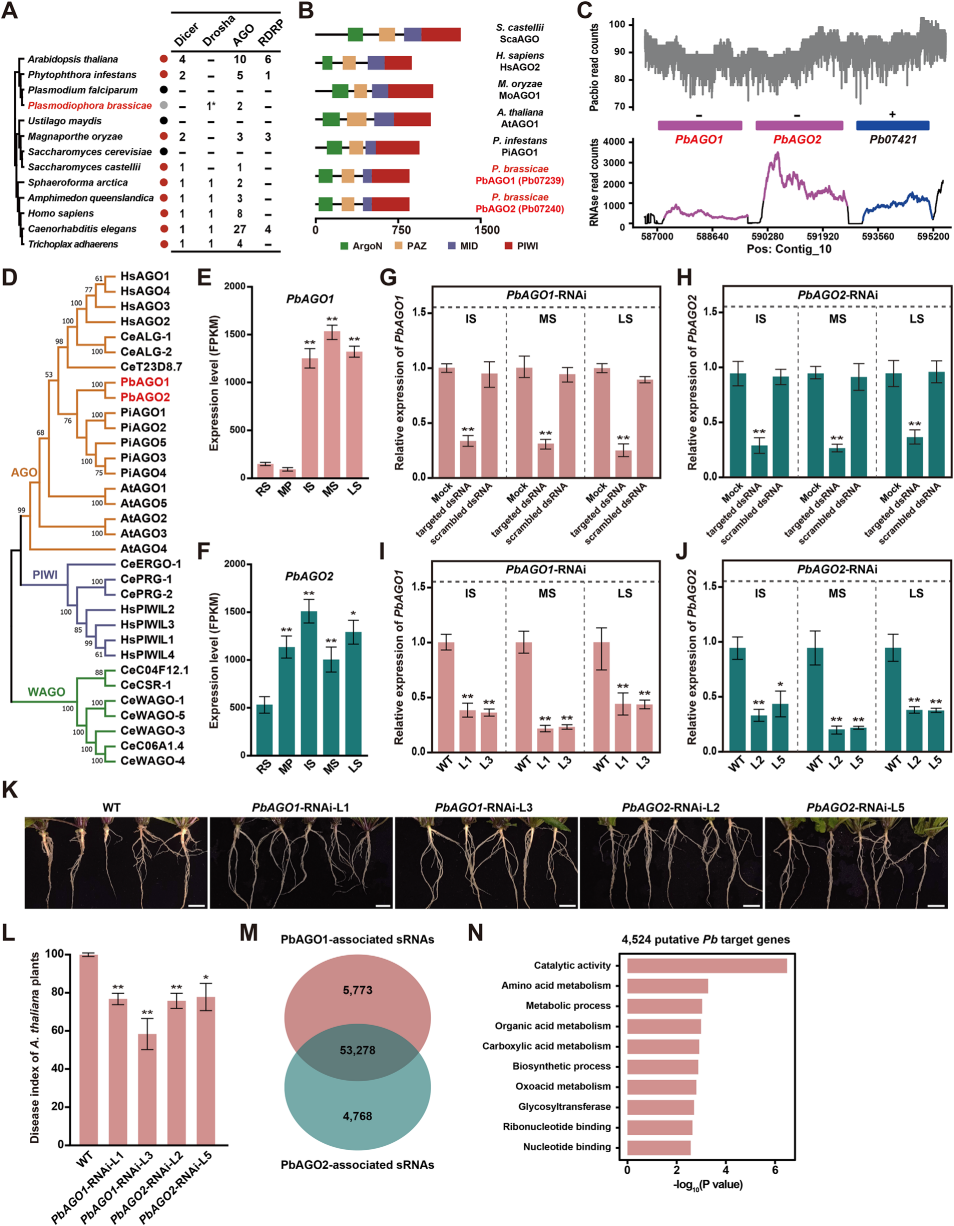
Identification and functional validation of two *Pb* Argonaute proteins. (A) Identification of the core RNAi pathway components in *Pb*. The number of identified RNAi core components is indicated. The species tree was obtained from the TimeTree5 web server (http://www.timetree.org/) and visualized using MEGA X software. Species with or without a functional RNAi pathway are marked with red or black circles, respectively. *indicates a non‐canonical Drosha homolog;—indicates the absence of recognizable RNAi core components. (B) Domain architectures of PbAGO1, PbAGO2, and representative AGO proteins from diverse eukaryotic species. ArgoN, Argonaute N‐terminal domain; PAZ, Piwi/Argonaute/Zwille domain; MID, middle domain; PIWI, P‐element‐induced wimpy testis domain. The scale bar indicates protein and domain sizes. (C) Validation of the *PbAGO1* and *PbAGO2* gene models by deep sequencing. The frequencies of PacBio long reads and Illumina short reads matching the genomic regions and transcripts of *PbAGO1* and *PbAGO2* are shown. (D) Phylogenetic analysis of AGO proteins from representative eukaryotic species. The tree was generated in MEGA X using the Jones–Taylor–Thornton (JTT) model based on the full‐length amino acid sequences of 33 AGO proteins. Bootstrap values >50% are indicated at the nodes. Branches are color‐coded to denote different AGO groups: orange, AGO; indigo, WAGO; green, PIWI. Hs, *Homo sapiens*; Ce, *Caenorhabditis elegans*; Pb, *P. brassicae*; At, *Arabidopsis thaliana*; Pi, *Phytophthora infestans*. (E,F) Expression profiles of *PbAGO1* and *PbAGO2* across the five *Pb* life stages, as revealed by transcriptome sequencing. (G,H) Functional verification of *PbAGO1* and *PbAGO2* in gene silencing through exogenous dsRNA treatment. (I,J) Functional verification of PbAGO1 and PbAGO2 in gene silencing through expression of endogenous sRNAs targeting PbAGO1 and PbAGO2 in transgenic Arabidopsis plants. (K,L) Effects of *PbAGO1* or *PbAGO2* silencing on *Pb* pathogenicity. Roots of *Arabidopsis* wild‐type (WT), *PbAGO1*‐RNAi, and *PbAGO2*‐RNAi plants inoculated with *Pb* were harvested at 20 dpi. Scale bars in K = 1 cm. (M) Venn diagram of *Pb* sRNAs associated with PbAGO1 and PbAGO2. (N) Gene Ontology (GO) enrichment analysis of sRNA target genes associated with PbAGO1 and PbAGO2. Significant top 10 items were plotted. Data in (E–J) and (L) are presented as mean ± SD. Statistical significance was determined by two‐sided Student's *t*‐tests using three biological replicates. *p < 0.05, **p < 0.01.

The identification of functional *Pb* siRNAs and miRNAs prompted us to further investigate the potential RNAi pathway in *Pb*. To this end, we searched the assembled *PbJS* genome for homologs of the canonical RNAi core components, including Dicer, Drosha, AGO, and RdRP proteins. As a result, we identified two canonical AGO homologs and one Drosha homolog, but no recognizable homologs of Dicer or RdRP (Figure 3A). Domain architecture analysis showed that the two identified *Pb* AGO homologs contain the four typical domains of canonical eukaryotic AGOs and these proteins were therefore designated PbAGO1 (Pb07239) and PbAGO2 (Pb07240), respectively (Figure 3B). More than 85‐fold PacBio long‐read coverage and 200‐fold Illumina read coverage across the genomic regions of PbAGO1 and PbAGO2 confirmed the accuracy of their assembly and annotation. Interestingly, *PbAGO1* and *PbAGO2* are arranged in tandem in the *Pb* genome (Figure 3C). Phylogenetic analysis further showed that, among members of the AGO superfamily from representative species, PbAGO1 and PbAGO2 cluster within the AGO family rather than the PIWI or WAGO subfamilies (Figure 3D). Multiple sequence alignment of the PIWI domains revealed that both PbAGO1 and PbAGO2 contain the conserved DDH catalytic residues characteristic of slicer‐active AGO proteins (Figure S5). Transcriptome sequencing showed that both PbAGO1 and PbAGO2 are highly expressed at the IS, MS, and LS stages, but are expressed at relatively low levels at the RS stage (Figure 3E,F), suggesting potential roles in *Pb* secondary infection.

To clarify the functions of PbAGO1 and PbAGO2 in gene silencing, we prepared sequence‐specific dsRNA fragments (targeted dsRNAs) and random dsRNA fragments (scrambled dsRNAs) based on the genomic sequences of PbAGO1 and PbAGO2, and used them to treat *Pb*‐inoculated *Arabidopsis* plants (Figure S6A). As expected, treatment with the targeted dsRNAs, but not the scrambled dsRNAs, resulted in marked downregulation of *PbAGO1* or *PbAGO2* expression relative to the mock treatment at all three examined life stages (Figure 3G,H). This result was further confirmed in the *Nicotiana benthamiana* system, in which exogenous application of targeted dsRNAs rather than scrambled dsRNAs reduced the expression of *PbAGO1* or *PbAGO2* compared with water treatment (Figure S7A,B). Correspondingly, dsRNA‐mediated silencing of *PbAGO1* or *PbAGO2* alleviated clubroot disease severity (Figure S7C,D). We also generated transgenic Arabidopsis plants expressing endogenous dsRNAs targeting PbAGO1 or PbAGO2 transcripts (Figure S6A). RT‐qPCR analysis showed that the expression of *PbAGO1* or *PbAGO2* in these transgenic plants was reduced to 55% or less of that in wild‐type plants at all three examined life stages (Figure 3I,J), accompanied by reduced disease severity (Figure 3K,L). Although the accumulation of *Pb* sRNAs indicated with *Pb‐miR041* was not obviously affected in the *PbAGO1*‐ or *PbAGO2*‐silenced transgenic plants, consistent with a previous study in Zebrafish [37], the expression of the *Pb‐miR041* target gene *Pb02485* was significantly increased at all three *Pb* life stages compared with that in wild‐type plants (Figure S7E,F).

To further determine whether PbAGO1 and PbAGO2 associate with endogenous Pb sRNAs, we successfully expressed both proteins (Figure S8A) and performed RNA immunoprecipitation sequencing (RIP‐seq). In total, 59 051 and 58 046 *Pb* sRNA species were recovered from the PbAGO1‐ and PbAGO2‐immunoprecipitated sRNA pools, respectively. Of these, 53 278 sRNA species were shared between the two datasets, whereas only 5773 and 4768 sRNA species were specific to PbAGO1 and PbAGO2, respectively (Figure 3M and Figure S9A–C), suggesting that PbAGO1 and PbAGO2 do not exhibit obvious binding preferences for distinct sRNA populations. For the sequenced 53 278 sRNA species associated with both PbAGO1 and PbAGO2, 4524 target genes were predicted and subjected to Gene Ontology (GO) enrichment analysis (Figure 3N). The results showed that these targets are involved in diverse biological processes, including catalytic activity, amino acid metabolism, organic acid and carboxylic acid metabolism (Figure 3N). Collectively, these results demonstrate that *Pb* possesses two canonical and functional AGO proteins.

### 2.4 A Drosha‐Like Protein is Required for sRNA Biogenesis in *Pb*

**FIGURE 4:**
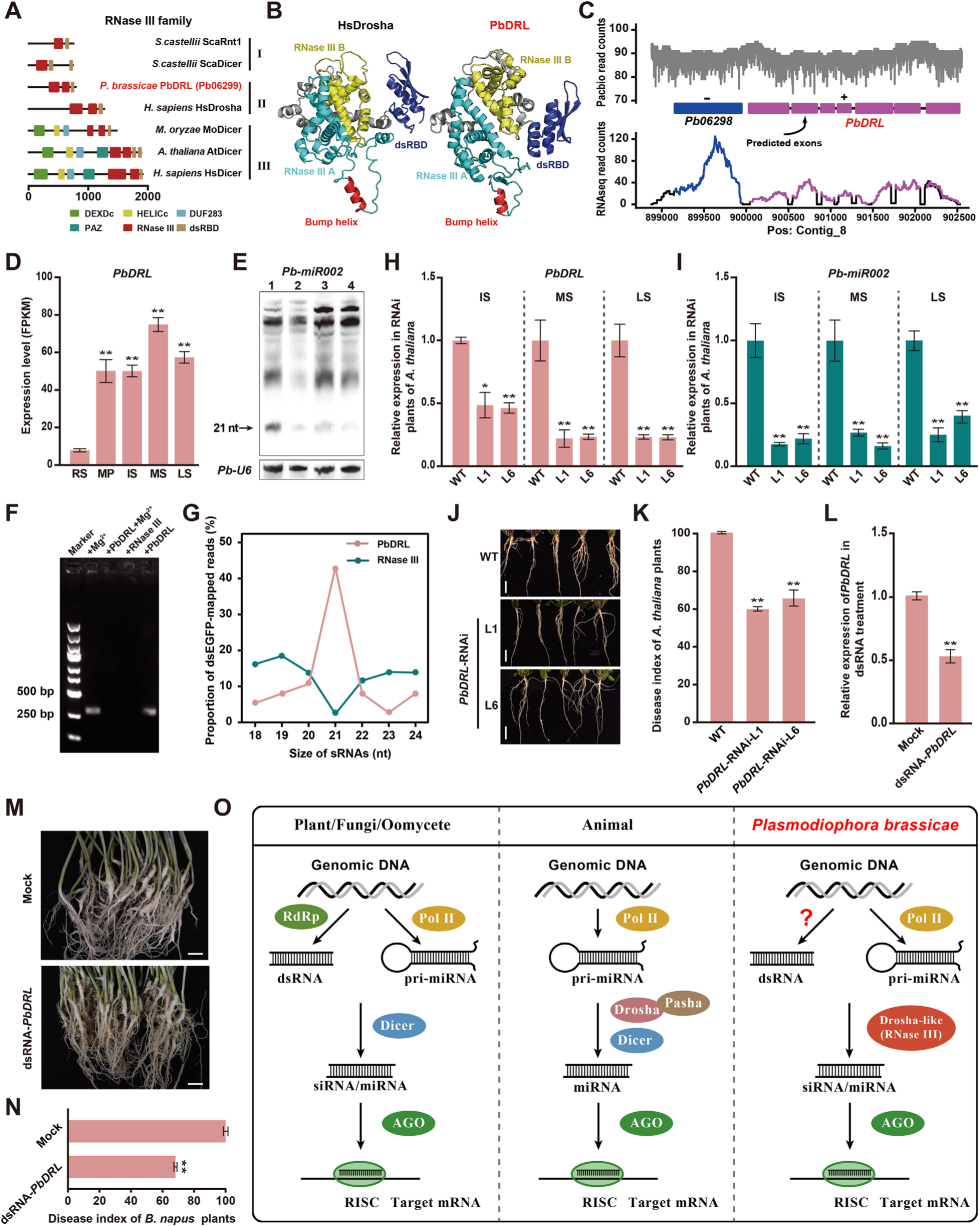
Functional validation of PbDRL in sRNA biogenesis. (A) Domain architectures of three types of eukaryotic RNase III proteins. Conserved domains are indicated: DEXD, DEAD‐like helicase superfamily domain; HELIC, helicase superfamily C‐terminal domain; DUF, DUF283 domain; RNase III, ribonuclease III domain; dsRBD, double‐stranded RNA‐binding domain. (B) Comparison of the predicted 3D structures of HsDrosha and PbDRL. The Drosha‐specific “Bump” helix is highlighted in red. (C) Validation of the *PbDRL* gene model by deep sequencing. Shown are the frequencies of PacBio long reads and Illumina short reads mapped to the genomic region and transcript of *PbDRL*. (D) Expression levels of *PbDRL* at five life stages based on transcriptome sequencing. (E) Validation of PbDRL function in sRNA biogenesis by Northern blot analysis. dsRNA mimics targeting *PbDRL* were incubated with resting spore suspensions, and total RNA was isolated for Northern blot detection. *Pb* U6 snRNA was used as an internal control. This experiment was repeated three times with similar results. (F) In vitro enzymatic assay of PbDRL for dsRNA cleavage activity. An EGFP dsRNA fragment was used as the substrate for this assay. RNase III was used as a control (Beyotime Biotechnology, China). (G) Small RNA sequencing of the products generated after PbDRL or RNase III cleavage of the dsRNA substrate. (H) Silencing of *PbDRL* by expression of sRNAs in transgenic *Arabidopsis* plants, and analysis of *PbDRL* expression at three life stages during *Pb* infection. (I) Effects of *PbDRL* silencing on representative sRNA production in transgenic *Arabidopsis* plants during *Pb* infection, as determined by stem‐loop RT‐qPCR. (J,K) Effects of *PbDRL* silencing on *Pb* pathogenicity in transgenic *Arabidopsis* plants expressing sRNAs. Infected roots were collected at 20 dpi. Scale bars in J = 1 cm. (L–N) Effects of *PbDRL* silencing by dsRNA treatment on *Pb* pathogenicity in *B. napus* plants. Infected roots were collected at 30 dpi. Scale bars in M = 1 cm. (O) Proposed models of RNAi pathways in plants/fungi/oomycetes, animals, and *Pb*. In plants, fungi, and oomycetes, siRNAs are generated from dsRNA synthesized by RNA‐dependent RNA polymerase (RdRP) and then cleaved by Dicer, whereas miRNAs are generated from pri‐miRNAs transcribed by RNA polymerase II (Pol II) and then cleaved by Dicer. In animals, only miRNAs are produced; they originate from pri‐miRNAs transcribed by Pol II and are sequentially processed by the Drosha–Pasha complex and Dicer. In *Pb*, the source of dsRNA for the siRNA pathway remains unknown. For the miRNA pathway, we propose that pri‐miRNAs are transcribed by Pol II and processed by a putative Drosha‐like protein. In all cases, mature siRNAs or miRNAs are loaded into Argonaute (AGO) proteins to form the RNA‐induced silencing complex (RISC), which mediates cleavage of target mRNAs. Data in (D, E, H, I, K, L, and N) are presented as mean ± SD. Statistical significance was determined by two‐sided Student's *t*‐tests with three biological replicates. *p < 0.05, **p < 0.01.

After characterizing the AGO proteins in the *Pb* RNAi pathway, we next sought to identify the proteins involved in sRNA biogenesis in *Pb*. Analysis of the PbJS genome together with other publicly available *Pb* genomes [38] identified only one Drosha homolog, Pb06299, but no Dicer homolog (Figure 3A). Pb06299 contains the characteristic domain architecture of Drosha proteins, including two RNase III domains followed by a dsRNA‐binding domain (dsRBD) (Figure 4A), and clusters with Drosha proteins in sequence‐based phylogenetic analyses (Figures S10A and S11A). Importantly, the predicted 3D structure of Pb06299 is highly similar to that of structurally resolved human Drosha (HsDrosha) and also contains the Bump helix motif (Figure 4B and Figures S10B and S11B), a conserved feature of Drosha proteins that forms one side of the RNA‐binding ravine near the basal junction [39]. Deep sequencing with at least 70‐fold PacBio long‐read coverage and 20‐fold Illumina read coverage further confirmed the accuracy of the Pb06299 gene assembly and annotation (Figure 4C). We therefore designated Pb06299 as *Plasmodiophora brassicae* Drosha‐like protein (PbDRL), identifying it as the primary candidate responsible for sRNA biogenesis in *Pb*.

To verify the role of PbDRL in *Pb* sRNA biogenesis, we first synthesized the dsRNA mimics targeting *PbDRL* (Figure S6A), and used them to incubate the purified resting spore suspensions at 28°C for 12, 18, or 24 h. Total RNA was then isolated from resting spores before and after dsRNA treatment for Northern blot analysis of *Pb* sRNA accumulation. The result showed that compared to the mock treatment, dsRNA treatment markedly reduced the accumulation of Pb‐miR002 in resting spores (Figure 4E). We next expressed and purified PbDRL protein for an in vitro enzymatic assay to test its dsRNA cleavage activity (Figure S8B). An EGFP dsRNA fragment was prepared as the substrate for this assay. We found that PbDRL exhibits a high dsRNA cleavage activity under Mg^2^^+^ conditions and is able to efficiently degrade the dsRNA substrate to undetectable levels, comparable to the activity of RNase III (Beyotime Biotechnology, China) (Figure 4F). In contrast, no detectable enzymatic activity was observed under Mg^2^^+^‐free conditions (Figure 4F). Furthermore, sRNA sequencing of the cleavage products generated by PbDRL from EGFP dsRNA revealed a predominant 21‐nt sRNA population, whereas the products generated by RNase III ranged from 18 to 24 nt in length (Figure 4G). In *Pb*‐inoculated transgenic Arabidopsis plants expressing endogenous dsRNAs targeting PbDRL transcripts, successful silencing of PbDRL led to a significant reduction in *Pb* miRNA production across all three examined life stages, as indicated by Pb‐miR002 levels (Figure 4H,I), further confirming the essential role of PbDRL in *Pb* sRNA biogenesis. In animals, Drosha‐mediated dsRNA processing requires two dsRBD‐containing proteins to form a heterotrimeric complex [6]. We therefore searched the *PbJS* genome for proteins containing dsRBD domains and identified two candidates, Pb09092 and Pb07475, each harboring three dsRBD domains (Figure S12A). However, yeast two‐hybrid assays did not detect their interactions with PbDRL (Figure S12B), suggesting that PbDRL may function through a distinct sRNA biogenesis mechanism in *Pb*. Moreover, both host‐induced and exogenous dsRNA‐mediated silencing of *PbDRL* compromised *Pb* infection and alleviated disease symptoms (Figure 4J–N). Collectively, these results demonstrate that the RNase III protein PbDRL is required for sRNA biogenesis in *Pb*.

### 2.5 Exploring RNAi Targets During *Pb* Early Infection

**FIGURE 5:**
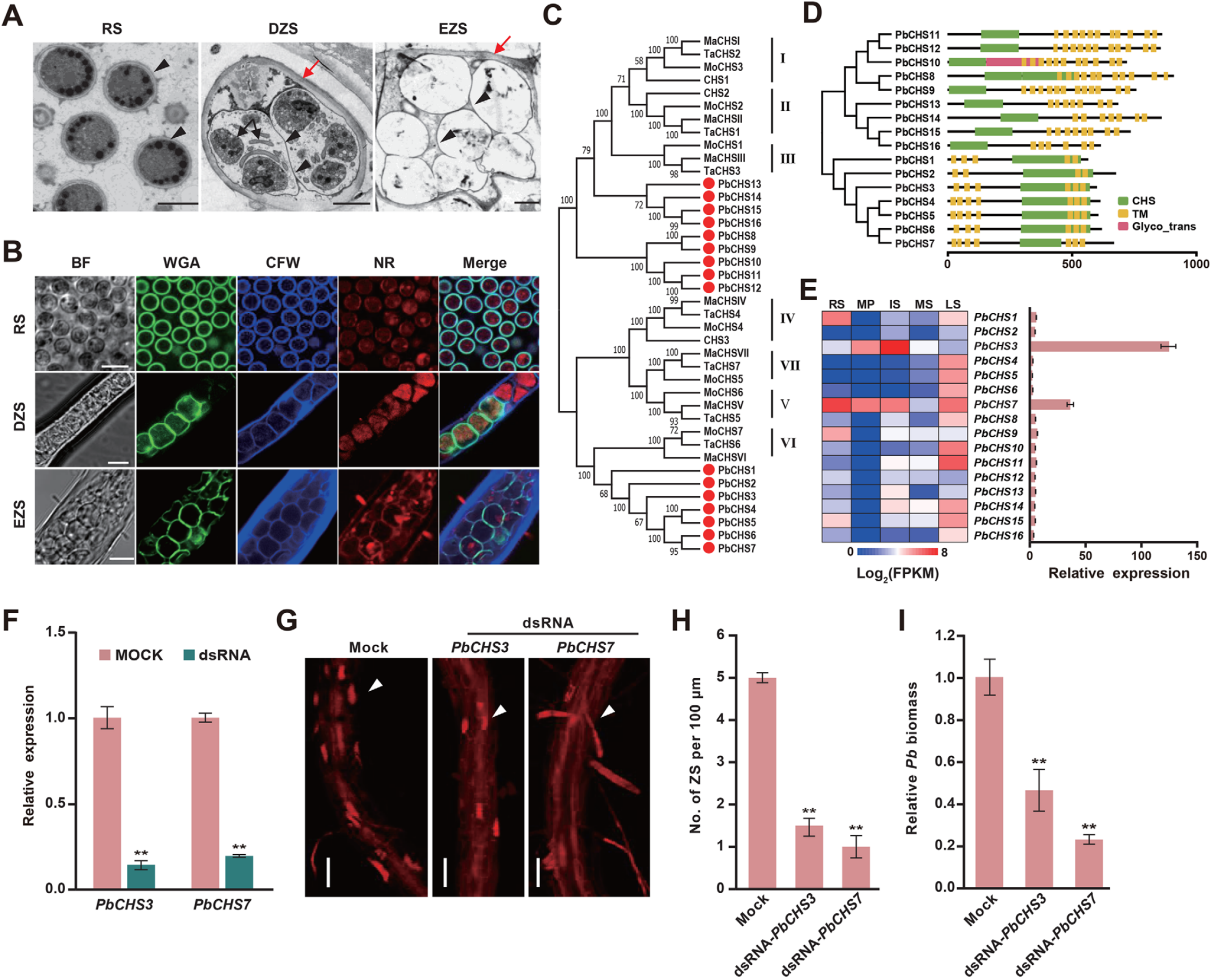
Identification of chitin and chitin synthase genes required for the development of *Pb* zoosporangia. (A) A wall shell present in zoosporangia revealed by TEM. Root tissues containing developing zoosporangia (DZS), empty zoosporangia (EZS), and resting spores (RS) were collected at 4, 6, and 24 dpi, respectively. Scale bars = 5 µm. (B) Identification of chitin in the zoosporangial wall shell. Wheat germ agglutinin (WGA) conjugated with Alexa Fluor 488 and the fluorescent dye calcofluor white (CFW) were used to stain chitin, whereas Nile red (NR) was used to stain *Pb* lipid droplets. Scale bars = 10 µm. (C) Maximum‐likelihood phylogeny of *Pb* chitin synthases (CHSs). The phylogenetic tree was generated using MEGA X with the JTT model based on the full‐length amino acid sequences of 40 CHSs. Nodes with bootstrap support >50% are indicated. Seven established CHS groups (I–VII) within the fungal kingdom are shown. (D) Domain architectures of 16 *Pb* CHSs. CHS, chitin synthase domain; TM, transmembrane domain; Glyco_trans, glycosyltransferase domain. The scale bar indicates protein and domain sizes. E, Expression levels of PbCHS1–16 at different life stages revealed by transcriptome sequencing and RT–qPCR. F, Expression levels of PbCHS3 and PbCHS7 in Pb‐inoculated Arabidopsis plants at 6 dpi before and after dsRNA treatment. (G,H) Effects of individual silencing of *PbCHS3* and *PbCHS7* on zoosporangial development in root tissues. Zoosporangia were labeled with Nile red and observed by confocal microscopy at 6 dpi. Scale bars in G = 10 µm. (I) Effects of individual silencing of *PbCHS3* and *PbCHS7* on *Pb* biomass in the roots of dsRNA‐treated *Arabidopsis* plants at 20 dpi. Data in (E, F, H, and I) are presented as mean ± SD from three biological replicates. Statistical significance was determined by a two‐tailed Student's *t*‐test, *p < 0.05, **p < 0.01.

The presence of a functional RNAi pathway in *Pb* prompted us to explore RNAi‐based strategies for controlling clubroot disease. During the *Pb* life cycle, many different life forms are developed, and only resting spores (RS) have previously been confirmed to possess a cell wall [17, 20], with chitin as a major structural component [40]. Chitin is widespread in fungi and insects as a structural component but is absent in plants, thereby becoming a specific and eco‐friendly target for control of various fungal diseases and insect pests [41, 42]. However, because RS are formed at the end of the *Pb* life cycle when root clubs have been developed (Figure 1A), targeting RS chitin would be too late to effectively control clubroot disease. We therefore re‐examined the *Pb* life cycle, with particular attention to early infection stages. Transmission electron microscopy (TEM) revealed a thin wall surrounding each subspherical zoosporangium, and it can be left in root epidermal cells after the release of secondary zoospores (Figure 5A). To determine whether the zoosporangial wall contains chitin, we performed immunofluorescence assays using wheat germ agglutinin (WGA), a chitin‐specific probe conjugated to Alexa Fluor 488. Strong green fluorescence signals were detected on the surface of both immature and empty zoosporangia, comparable to those observed in resting spores (Figure 5B). This finding was further confirmed by calcofluor white (CFW) staining, which binds chitin and cellulose (Figure 5B), demonstrating that chitin is a structural component of the zoosporangial wall.

Analysis of the assembled *PbJS* genome identified 16 putative chitin synthase genes (*PbCHS1–16*), all containing the characteristic domains of the CHS family and clustering into two distinct phylogenetic clades (Figure 5C,D). Life stage‐specific transcriptome analysis showed that *PbCHS3* and *PbCHS7* were highly expressed at the middle primary infection (MP) stage, when zoosporangia are forming, and their expression patterns were further validated by RT–qPCR (Figure 5E). These results suggest that *PbCHS3* and *PbCHS7* are likely involved in chitin biosynthesis of the zoosporangial wall. Because *Pb* is an obligate parasite, gene silencing was used to assess the functions of *PbCHS3* and *PbCHS7* in zoosporangial wall chitin biosynthesis. Silencing either *PbCHS3* or *PbCHS7* by exogenous dsRNAs in *Pb*‐inoculated *Arabidopsis* plants significantly reduced zoosporangia formation in root epidermal tissues and decreased *Pb* biomass (Figure 5F–I and Figure S6A). Together, these findings identify chitin and its biosynthetic genes during *Pb* early infection as promising RNAi targets for the control of clubroot disease.

### 2.6 Exploring RNAi to Control the Clubroot Disease

**FIGURE 6:**
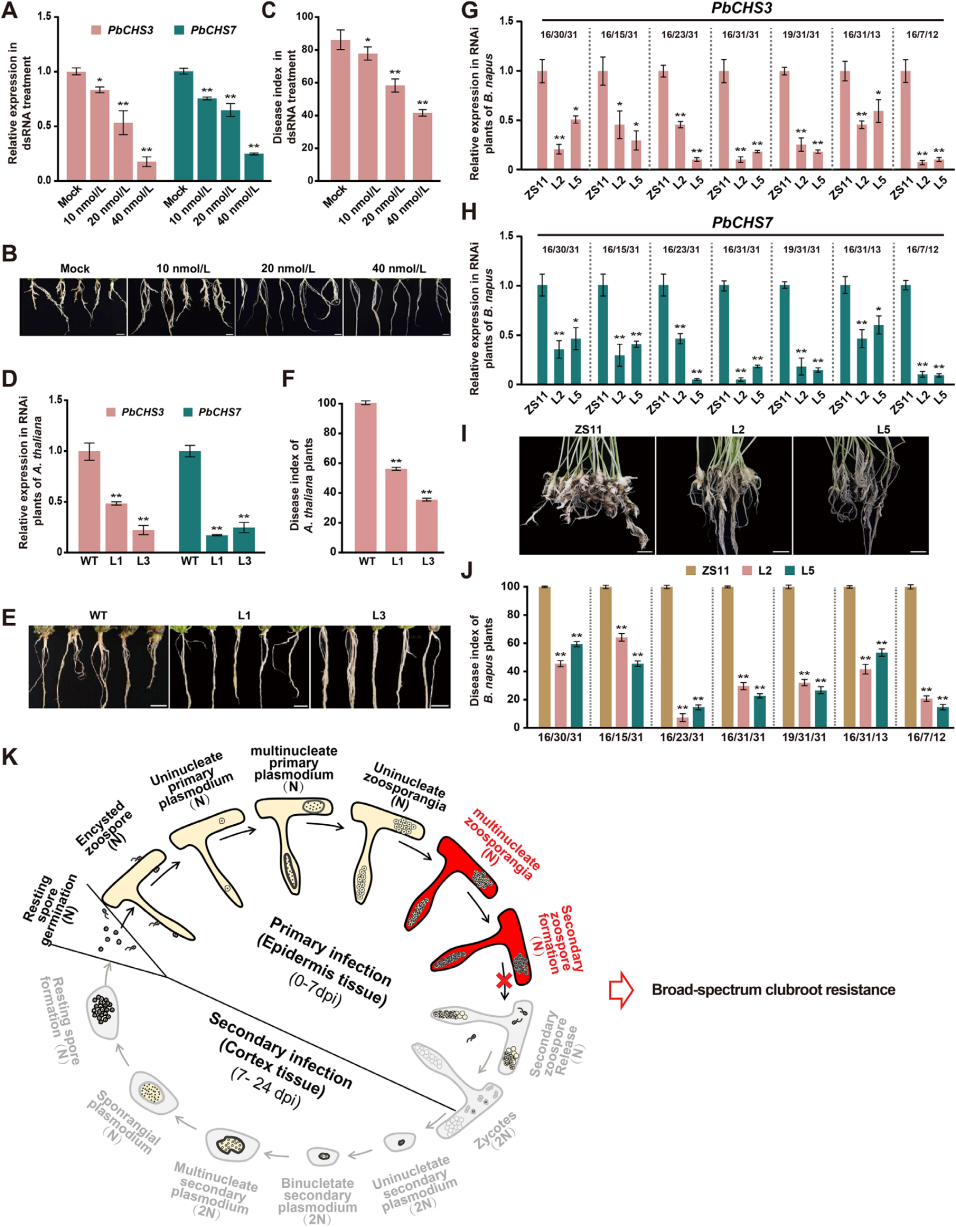
Evaluation of *PbCHS3* and *PbCHS7* silencing for disease control. (A–C) Exogenous dsRNA‐mediated silencing of *PbCHS3* and *PbCHS7* significantly reduced clubroot disease severity in *Arabidopsis* plants. *Pb*‐infected plants were harvested at 7 dpi to examine *PbCHS3* and *PbCHS7* expressions, and at 20 dpi to evaluate disease severity. Scale bars in B = 1 cm. (D–F) Host‐induced gene silencing of both *PbCHS3* and *PbCHS7* significantly enhanced clubroot resistance in transgenic *Arabidopsis* plants. *Pb*‐infected *Arabidopsis* plants were harvested at 20 dpi. Scale bars in E = 1 cm. (G–J) Host‐induced gene silencing of both *PbCHS3* and *PbCHS7* conferred broad‐spectrum clubroot resistance in transgenic *B. napus* plants. Seven different pathotypes were included. *Pb*‐infected *B. napus* roots were harvested at 30 dpi. Scale bars in I = 2 cm. (K) Proposed model showing that interference with chitin biogenesis during *Pb* zoosporangial development, achieved through host‐induced gene silencing of *PbCHS3* and *PbCHS7*, can confer broad‐spectrum clubroot resistance. The *Pb* life cycle model was adapted from our previous study [17], and the *Pb* life stages targeted by RNAi are indicated in red. Data in (A, C, D, F, G, H, and J) are presented as mean ± SD. Statistical significance was determined by a two‐sided Student's *t*‐test using three biological replicates. *P < 0.05, **P < 0.01.

To evaluate the potential of silencing *PbCHS3* and *PbCHS7* for clubroot disease control, we selected a conserved sequence fragment shared by both genes for dsRNA synthesis and vector construction (Figure S6B). Exogenous application of the dsRNA preparation effectively silenced both *PbCHS3* and *PbCHS7* in *Pb*‐inoculated plants and reduced disease severity, with the disease index decreasing from 84 to 42 at a dsRNA concentration of 40 nmol/L (Figure 6A–C). We next generated transgenic Arabidopsis and *B. napus* plants expressing the same conserved DNA fragment to produce endogenous dsRNAs targeting both PbCHS3 and PbCHS7 transcripts. As expected, these transgenic plants efficiently suppressed the expression of both genes during *Pb* infection and showed strong potential to enhance plant resistance and reduce clubroot disease severity (Figure 6D–F). Furthermore, testing against seven different *Pb* pathotypes showed that the transgenic *B. napus* plants were resistant to all seven pathotypes [25], indicating broad‐spectrum resistance to clubroot disease (Figure 6G–J). Therefore, we identify the chitin biosynthesis of zoosporangial wall as a new target and RNAi interfering with this process is a promising strategy for controlling the clubroot disease.

## 3 Discussion

Although Rhizaria represents a unique eukaryotic taxonomic group and a fascinating reservoir of highly diverse microbes, its molecular basis remains poorly understood. To our knowledge, this study provides the first systematic characterization of the RNAi landscape across multiple Rhizaria species and reveals both the conserved and unique molecular features of RNAi in *Pb* compared with established RNAi model systems. Similar to plants, fungi, and oomycetes, *Pb* produces two major classes of small RNAs, namely siRNAs and miRNAs (Figure 2F), and possesses functional canonical AGO proteins (Figures 3 and 4O). Interestingly, however, the core RNAi component RdRp, which is responsible for generating the long dsRNA precursors of siRNAs in plants, fungi, and oomycetes, is absent in *Pb* (Figures 3A and 4O). How siRNA precursors are generated in *Pb* therefore remains unclear, although PbDRL is able to directly process long dsRNAs into siRNAs (Figure 4F,G). One hallmark feature of the *Pb* RNAi pathway revealed by this study is that *Pb* uses a Drosha‐like protein, PbDRL, rather than canonical Dicer proteins, to process long dsRNAs or pri‐miRNAs into mature siRNAs or miRNAs (Figure 4). This feature is highly unusual and distinct from all previously characterized RNAi models, because Drosha proteins are mainly found in animals and are generally responsible only for pri‐miRNA processing rather than direct production of mature miRNAs [6]. In animals, Drosha requires two dsRBD‐containing partner proteins to form a heterotrimeric complex for pri‐miRNA processing [6]. In *Pb*, however, our evidence showed that PbDRL does not interact with the two identified dsRBD‐containing proteins, and that PbDRL itself possesses dsRNA cleavage activity (Figure 4F,G and Figure S12). The mechanism by which PbDRL mediates sRNA biogenesis in *Pb* is therefore still not fully understood. Overall, our study provides new insights into RNAi in Rhizaria and expands our understanding of RNAi diversity across eukaryotes.

Notably, non‐canonical pathways for sRNA biogenesis have also been reported in other eukaryotes. In the protozoan parasite *Toxoplasma gondii*, a representative member of the SAR supergroup that is phylogenetically closely related to *Pb*, a non‐canonical Dicer‐like protein, Tg‐Dicer, has been reported to likely generate both siRNAs and miRNAs, although it shows substantial divergence in primary sequence and domain architecture and lacks the dsRBD domain [43]. In zebrafish and mice, which possess multiple AGO proteins, AGO2 rather than Dicer is responsible for the biogenesis of mature miR‐451 [8, 37]. In addition, PIWI‐interacting RNAs (piRNAs), a distinct class of small RNAs, have been identified in animals and are typically characterized by a 10‐nt 5′‐to‐5′ overlap [44]. However, the predominant 19‐nt interval observed between *Pb* sRNAs mapped to the plus and minus strands of the same genomic loci does not support the existence of piRNA biogenesis in *Pb* (Figure 2B). Whether AGO proteins or other as‐yet‐unidentified factors participate in sRNA biogenesis in *Pb* therefore requires further investigation.

The clubroot disease caused by *Pb* is highly destructive to cruciferous crops and results in enormous economic losses worldwide. Although the identification and deployment of clubroot resistance genes for breeding remain major and cost‐effective strategies for disease control, the widespread emergence of diverse *Pb* pathotypes frequently undermines the resistance of previously resistant cultivars [24, 25, 45]. It is therefore urgent to develop new strategies and identify new targets for clubroot control. However, the life cycle of *Pb* is highly complex, with multiple distinct life forms developed sequentially during infection [17, 20]. As a result, the cellular and molecular characteristics of these different *Pb* life forms remain poorly understood, and potentially exploitable targets for disease control have so far rarely been reported. This challenge is further strengthened by the obligate intracellular parasitism of *Pb* and the limited genetic tools currently available for functional validation of *Pb* genes. Although a few previous studies have implied that RNAi may be effective in *Pb*, the RNAi pathway and its underlying mechanisms in this pathogen have remained entirely unknown. Our study clarifies the molecular basis of RNAi in *Pb* and demonstrates that RNAi can serve as a powerful tool for functional analysis of *Pb* genes. Importantly, we identified chitin in the zoosporangial cell wall during early *Pb* infection and demonstrated that this process represents a valuable and promising target for RNAi‐based or chemical control of clubroot disease in cruciferous crops (Figure 6K). Therefore, our study provides both a new target and a new tool for investigating and controlling clubroot disease.

## 4 Experimental Section

### 4.1 Plant Materials and Growth Conditions

All the Arabidopsis (*A. thaliana*) and *B. napus* plants were grown under standard growth conditions at 23°C with a16‐h light/ 8‐h dark photoperiod. The transgenic *PbAGO1*‐*RNAi*, *PbAGO2*‐*RNAi*, *PbDRL*‐*RNAi*, *PbCHS3*/7‐*RNAi* lines were generated in this study. All Arabidopsis plants were in the Columbia‐0 (Col‐0) genetic background, whereas *B. napus* plants were derived from the cultivar ZS11.

### 4.2 Pathogens, Inoculation, and Disease Assessment

*Pb* isolate *PbJS* was maintained on the susceptible *B. napus* cultivar Westar, and the clubbed roots were harvested at 30 dpi, dried in a forced‐air oven at 40°C, and stored at 4°C. To isolate and purify resting spores, the dried clubbed roots were rehydrated and homogenized in a blender. The resulting resting spore suspension was further purified as previously described [17]. Two‐week‐old Arabidopsis and *B. napus* plants were root‐inoculated with 1 mL resting spore suspension (1.0 × 10^7^ spores/mL), which was applied to the soil around the roots. Disease severity was assessed at 20 dpi for Arabidopsis and 30 dpi for *B. napus* plants using a standardized 0–3 grading system, and disease index was calculated as previously detailed [21]. The biomass of *Pb* in the roots of each material was quantified by qPCR using total DNA and the *Arabidopsis* actin gene (*AT3G18780*) was used as the internal control. The primers used here are listed in Table S10.

### 4.3 Genome Sequencing, Assembly, and Annotation

High‐purity resting spores of *PbJS* were used for genome sequencing by combining PacBio HiFi, Illumina and Hi‐C chromatin conformation capture technologies. The genomic DNA was extracted using a commercial DNA extraction kit (QIAGEN, China). Following the manufacturer's protocol, a high‐molecular‐weight genomic DNA library was prepared using the SMRTbell Express Template Prep Kit 2.0 (PacBio, USA) and sequenced on a PacBio Sequel II platform to generate long HiFi reads. In parallel, Illumina libraries were prepared using the Truseq Nano DNA HT Sample Preparation Kit (Illumina, USA), whereas Hi‐C libraries were constructed following the proximo Hi‐C protocol (Phase Genomics, USA). Both Illumina and Hi‐C libraries were sequenced on the Illumina HiSeq X platform using a 150 bp paired‐end strategy. Raw sequencing data were preprocessed with fastp v0.12.6 to remove adapter sequences and low‐quality reads [46].

To assess the genome size of *PbJS*, *K*‐mer frequency distribution was calculated using Jellyfish v2.2.3 based on the generated Illumina reads [47]. The resulting PacBio HiFi reads were assembled into raw contigs using Hifiasm v0.16.0 with default parameters [48]. The raw assemblies were iteratively polished using Illumina short reads. Finally, the paired‐end Hi‐C reads were mapped to the contigs using HiC‐Pro v3.1.0 [49], and the assembled genome was corrected with Juicebox v1.11.8. Benchmarking universal single‐copy orthologs (BUSCO) analysis with the Alveolata_odb10 database was employed to assess the genome assembly completeness of *PbJS*. Synteny analysis was performed using MCScanX with default parameters [50].

The repetitive sequences were annotated using a comprehensive transposable annotation pipeline EDTA v.2.1.0 with default parameters [51]. After repeat masking, protein‐coding genes were predicted using a combination of de novo, homology‐based, and transcriptome‐based approaches. The software Augustus v3.3.1 [52], GlimmerHMM v3.0.4 [53], and SNAP v2006‐07‐28 [54], were respectively used for de novo prediction of gene models. GeneWise v.2.2.0 was used for homology‐based prediction of gene models based on protein sequences from the reference genome of *Pb* isolate e3 and *P. falciparum* isolate 3D7 [55], a species with a phylogenetically closed relationship with *Pb*. The transcriptome reads were assembled into transcripts with Trinity v2.11.0 [56], which were further used for prediction of the gene structures by PASA v.2.3.3 [57]. Finally, all the predicted gene models by three approaches described above were incorporated by EVidenceModeler v1.1.1 to generate a consensus gene set [58].

### 4.4 Transcriptome Sequencing and Data Analysis

The purified resting spores, as well as *Pb*‐infected and ‐uninfected Arabidopsis roots at the MP, IS, MS and LS stages, were subject to transcriptome sequencing with three biological replicates. Transcriptome sequencing was performed on the Illumina HiSeq X platform using a 150‐bp paired‐end strategy. The raw Illumina reads were processed using fastp v0.12.6 to remove adapters and low‐quality bases [46]. The resulting high‐quality reads were simultaneously mapped to the *PbJS* reference genome and the Arabidopsis reference genome version 10 (TAIR10) (https://www.arabidopsis.org/) using Hisat2 v2.0.4 with default parameters [59]. The expression levels of the genes in each sample were estimated as fragments per kilobase per million mapped reads (FPKM) values, and differentially expressed genes (DEGs) between samples or conditions were identified using the R package DEseq2 v1.32.0. Genes with an adjusted *p*‐value < 0.05 and a fold change ≥ 2 were considered significantly differentially expressed.

### 4.5 sRNAome Sequencing, Identification, and Data Analysis

The same samples used for the transcriptome sequencing were also subjected to sRNAome sequencing with at least two biological replicates. Libraries were prepared according to the manufacturer's protocol. Subsequently, 50‐bp single‐end sequencing was performed on the Illumina platform. After quality control for adapter removal and the low‐quality read filtering, the clean reads were respectively mapped to the Rfam v14.0 database and the Arabidopsis genome by Bowtie v1.0 to discard the rRNAs, tRNAs, snRNA, snoRNA, and host‐derived sRNAs [60]. The remaining reads that mapped to the *PbJS* reference genome with ≥10 counts were retained as *Pb* sRNAs for subsequent characterization. To annotate known miRNAs, *Pb* sRNAs were aligned against the miRBase v21.0 database (https://mirbase.org/) allowing no more than three mismatches relative to mature miRNA sequences. Novel miRNAs were predicted using ShortStack v3.8.2 with the following parameters: –strand_cutoff 0.5, –foldsize 1000, –dicermin 18, and –dicermax 38. Additionally, sRNAminer was employed to annotate phasiRNA‐like and hc‐siRNA‐like candidates using default settings. phasiRNA‐like candidates were identified using phasing score ≥ 10 and *p*‐value ≤ 0.001, whereas hc‐siRNA‐like candidates were defined by 23–24 nt sRNA enrichment and repeat‐associated features. GO terms were annotated using PANNZER2, and GO enrichment analysis of AGO‐associated target genes was performed with clusterProfiler. GO terms with an adjusted *p*‐value < 0.01 were considered significantly enriched.

The expression levels of sRNAs in each sample were quantified as reads per million mapped reads (RPM). The frequency of distances separating all 21‐nt sRNA 5′‐end pairs mapping to the same DNA strand was calculated using the following equation: Frequency(*D*) =$\sum_{i,j}\left(readi\;readj\right)D\;$, where *D* represents the distance between the 5′ ends of *read_i_* and *read_j_*. Similarly, the frequency of distances separating all 21‐nt sRNA 5′‐end pairs mapping to opposite DNA strands was calculated separately using the same equation. To examine the ping‐pong signature, 5′–5′ overlap distributions of opposite‐strand *Pb* sRNA pairs were calculated for 18–30 nt and 21‐nt sRNAs, with overlap frequencies weighted by RPM‐normalized pooled sRNA abundance. The secondary structures of the miRNA precursor genomic sequences were visualized using RNAfold v2.1.9. miRNA targets in *Pb* were predicted by miRanda v3.3a with a score threshold of >100 and an energy threshold of < −23 kcal/mol.

### 4.6 RT‐qPCR and Northern Blot Analysis

The expression levels of selected genes were determined as previously described [61]. The expression levels of miRNAs were quantified by stem–loop RT‐qPCR as previously described [62]. First‐strand cDNA was synthesized using miRNA First Strand cDNA Synthesis (Sangon, China), and PCR detection was performed using a miRNA‐specific forward primer and a universal reverse primer. *Pb* U6 snRNA was used as the internal control for *Pb* miRNA quantification, whereas *B. napus* U6 snRNA was used as the internal control for *B. napus* miRNA quantification. The primers used for RT‐qPCR analysis are listed in Table S10. For Northern blot analysis, 5 µg total RNA was resolved on denaturing 15% polyacrylamide gels containing 7 M urea and subsequently transferred to Hybond‐N+ membranes (Cytiva, USA). Hybridization was conducted at 58°C using biotin‐labeled locked nucleic acid (LNA)‐modified DNA probes. Complete probe sequences are provided in Table S11.

### 4.7 dsRNA Synthesis and Treatment

Target sequences of *PbAGO1*, *PbAGO2*, *PbDRL*, *PbCHS3*, *PbCHS7*, and EGFP were amplified by PCR and transcribed into dsRNA using the T7 RNAi Transcription Kit (Vazyme, China). EGFP‐derived dsRNA was used as the dsEGFP substrate for the in vitro PbDRL cleavage assay. Scrambled dsRNA controls for PbAGO1 and PbAGO2 were generated from commercially synthesized DNA templates containing scrambled sequences (Genecreate, China) and transcribed using the same T7‐based method. Purified dsRNAs were resuspended in RNase‐free water. For soil drenching, dsRNAs were diluted to final concentrations of 10, 20, and 40 nmol/L and mixed with amino‐functionalized dendritic mesoporous silica nanoparticles (XFNANO, China) and RNase Inhibitor. Aliquots were cryopreserved at −80°C and thawed on ice for 30 min before application. Soil drenching was performed at 3‐day intervals from 5 to 20 dpi, with 1 mL of dsRNA suspension applied to each plant per treatment. The primers used here are listed in Table S10.

### 4.8 Transient Expression Assay in *N. benthamiana*

The coding sequences of *PbAGO1* and *PbAGO2* were cloned into transient expression vectors and introduced into *Agrobacterium tumefaciens* strain GV3101. The resulting Agrobacterium suspensions were infiltrated into *N. benthamiana* leaves for transient expression. At 24 h after Agrobacterium infiltration, target‐specific dsRNAs or corresponding scrambled dsRNA controls were infiltrated into the same leaves. Leaf samples were collected at 36 h after dsRNA treatment, and the expression levels of *PbAGO1* and *PbAGO2* were quantified by RT‐qPCR. The primers used here are listed in Table S10.

### 4.9 Vector Construction and Plant Transformation

A hairpin RNAi cassette targeting *PbAGO1*, *PbAGO2*, *PbDRL*, *PbCHS3*, and *PbCHS7* (design schematics are shown in Figure S6) was engineered into the pBI121 backbone, resulting in the generation of the pBI121‐RNAi constructs. The integrity of the plasmid was verified by Sanger sequencing, and the confirmed constructs were transferred into *A. tumefaciens* strain GV3101. The resulting *Agrobacterium* cultures were used for floral‐dip transformation of Arabidopsis and Agrobacterium‐mediated transformation of *B. napus*. Transgenic plants were confirmed by PCR using gene‐specific primers and homozygous transgenic lines were used for subsequent phenotypic and expression analyses. The primers used here are listed in Table S10.

### 4.10 Confocal Laser Scanning Microscopy and Transmission Electron Microscopy

*Pb*‐infected Arabidopsis roots were collected at 4–7 dpi for zoosporangia observation and at 24 dpi for resting spore analysis. The roots were thoroughly rinsed to remove soil particles, gently dried, and fixed in formalin‐acetic acid‐alcohol (FAA) to preserve structural integrity. Triple fluorescent staining was performed with wheat germ agglutinin (WGA) for chitin labeling, Calcofluor White (CFW) for chitin detection, and Nile Red (NR) for lipid droplets. Excitation wavelengths and emission filter settings for WGA, CFW, and NR were set according to a previous method [17]. Confocal Laser Scanning Microscopy (CLSM) was performed using a Zeiss LSM Meta 510 confocal microscope (Carl Zeiss, Germany), with contrast and brightness settings optimized to achieve high‐resolution imaging and detailed structural visualization of *Pb*. Transmission Electron Microscopy (TEM) analysis was performed as previously described [23].

### 4.11 Validation of miRNA‐Mediated Target Silencing

The cleavage activity of *Pb* miRNAs (*Pb‐miR002*, *Pb‐miR041*, *Pb‐miR045*) on their putative targets (*Pb05050*, *Pb04285*, *Pb07374*) was validated using a dual‐luciferase reporter assay. miRNA sequences synthesized in BioRun Tech. (Wuhan, China) were cloned into the pGreenII‐62SK effector vector, while wild‐type and mutated target fragments (containing disrupted miRNA‐binding sites) were inserted into the pGreenII0800 reporter vector. Arabidopsis protoplasts were co‐transfected with effector‐reporter plasmid pairs, with the empty pGreenII‐62SK vector serving as the negative control. Luminescence signals were quantified using the Dual‐Luciferase Reporter Assay System (Promega, USA), with firefly luciferase activity normalized to Renilla luciferase activity to correct for transfection efficiency.

### 4.12 Expression, Purification, and Immunoprecipitation of PbAGO Proteins

The coding sequences of PbAGO1 and PbAGO2 were amplified and cloned into engineered pMlink vectors with a FLAG tag. The tag position was indicated by the construct name: PbAGO1‐FLAG and PbAGO2‐FLAG indicate C‐terminally FLAG‐tagged proteins, whereas FLAG‐PbAGO1 and FLAG‐PbAGO2 indicate N‐terminally FLAG‐tagged proteins. Expi293F cells transfected with PbAGO constructs were incubated for 60 h. Cells were harvested, washed with PBS, and lysed in buffer containing 50 mm Tris‐HCl, pH 7.4, and 150 mm NaCl. After high‐pressure cell disruption, the lysate was clarified by centrifugation at 15 000 g for 30 min and incubated with anti‐FLAG affinity resin for 1 h. The resin was washed with the same buffer, and bound proteins were eluted with buffer supplemented with 150 µg/mL FLAG peptide. The eluted proteins were used for subsequent biochemical analyses.

For AGO‐associated sRNA sequencing, lysates containing FLAG‐tagged PbAGO1 or PbAGO2 complexes were incubated with Flag‐Trap beads at 4°C for 4 h. After three washes with 500 µL of washing buffer, RNAs associated with the immunoprecipitated PbAGO complexes were extracted from the beads and used for small RNA library construction and sequencing as described above. All buffers used for AGO‐associated RNA extraction were prepared under RNase‐free conditions. The primers used for vector construction are listed in Table S10.

### 4.13 Expression and Purification of PbDRL and In Vitro dsRNA Cleavage Assay

The coding sequence of PbDRL was amplified from the *Pb* cDNA library, cloned into a T‐cloning vector, and verified by sequencing. The sequence‐verified PbDRL fragment was subcloned into a modified pFastBac1 vector with an N‐terminal Strep tag and a C‐terminal His10 tag. Recombinant bacmids were generated in DH10Bac cells according to the manufacturer's instructions for the Bac‐to‐Bac baculovirus expression system, and baculoviruses were generated and amplified in Sf9 insect cells. High Five (*Trichoplusia ni*) insect cells were infected with recombinant baculovirus expressing PbDRL and cultured at 27°C for 72 h. Cells were harvested and lysed in buffer containing 25 mm Tris‐HCl, pH 8.0, 150 mm NaCl, and 0.5 mm PMSF. After cell disruption and centrifugation at 20 000 g for 1 h at 4°C, the supernatant was loaded onto Strep affinity resin. Bound PbDRL was washed with lysis buffer and eluted with buffer containing 25 mm Tris‐HCl, pH 8.0, 150 mm NaCl, and 25 mm biotin.

Purified PbDRL protein was incubated with EGFP‐derived dsRNA (dsEGFP) to examine its dsRNA cleavage activity. Each 20 µL reaction contained 2 µL of 10× reaction buffer, 2 µL of purified PbDRL protein, 2 µg of dsEGFP substrate, and RNase‐free water. The 1× reaction buffer contained 20 mm Tris‐HCl, pH 7.5, 50 mm NaCl, 1 mm DTT, and 1 mm MgCl_2_. For Mg^2^^+^‐free controls, MgCl_2_ was omitted from the reaction buffer. Reactions were incubated at 37°C for 20 min and terminated by adding EDTA to a final concentration of 50 mm. Cleavage products were analyzed by electrophoresis on 4% agarose gels under RNase‐free conditions. Commercial RNase III (Beyotime, China) was used as a control where indicated. The primers used for vector construction are listed in Table S10.

### 4.14 Western Blot Analysis

Protein expression was verified by western blotting. Protein samples were separated by SDS‐PAGE and transferred to PVDF membranes. FLAG‐tagged PbAGO1 and PbAGO2 proteins were detected using an anti‐FLAG antibody, whereas Strep‐tagged PbDRL was detected using an anti‐Strep antibody.

### 4.15 Yeast Two‐Hybrid Assay

The coding sequence of *PbDRL* was cloned into the pGBKT7 vector as bait, whereas the coding sequences of *Pb09092* and *Pb07475* were cloned into the pGADT7 vector as prey. The bait construct was first tested for transcriptional self‐activation in *Saccharomyces cerevisiae* AH109. For interaction assays, bait and prey constructs were co‐transformed into yeast cells, and transformants were selected on SD‐Leu‐Trp medium. Protein interactions were further examined on SD‐Leu‐Trp‐His and SD‐Leu‐Trp‐His‐Ade media supplemented with X‐α‐Gal. Empty‐vector combinations were used as negative controls.

### 4.16 Identification of RNAi Pathway Components and Chitin Synthetases, Phylogenetic Analysis, and Structure Prediction

To identify core components of the RNAi pathway and chitin synthetases in *Pb*, reported RNAi pathway proteins (Table S12) and chitin synthetase sequences (Table S13) from representative species were used as queries to search the *Pb* protein database, with an E‐value threshold of < 1e‐5. Candidate sequences were subsequently validated through domain analysis using the InterProScan server (https://www.ebi.ac.uk/interpro). Multiple sequence alignments were generated using Muscle v5.1.0 with default settings [63], and visualized in JalView v2.11.4 [64]. Phylogenetic reconstruction was conducted using the maximum likelihood method under the Jones‐Taylor‐Thornton (JTT) substitution model in MEGA X [65], with branch support assessed through 1000 bootstrap replicates. The species tree was obtained from the TimeTree5 server (http://www.timetree.org/). Protein structures were clustered by the Dali server (http://ekhidna2.biocenter.helsinki.fi/dali/). Protein structure was predicted by AlphaFold v.2.1.1 with default settings [66]. The model with the highest predicted local distance difference test (pLDDT) score was selected for further analysis and visualized using PyMol v2.5.4 (https://pymol.org/).

### 4.17 Statistical Analysis

Data normalization was performed as described in the corresponding methods. Gene expression levels from transcriptome sequencing were normalized as FPKM values, sRNA abundance was normalized as RPM, RT‐qPCR data were calculated using the 2^−ΔΔCt^ method, and firefly luciferase activity was normalized to Renilla luciferase activity. Data are expressed as the mean ± standard deviation (SD) from three biological replicates unless otherwise stated. For disease assessment, each biological replicate included 30 plants. Statistical significance was determined by a two‐sided Student's *t*‐test. *p < 0.05 and **p < 0.01 were considered statistically significant. Statistical analyses were performed using GraphPad Prism and R.

## Author Contributions

L.J.L. and X.Z. conceived and designed the research. X.Z. analyzed the sequencing data and performed the bioinformatic analysis; Y.P.W. and F.G. performed northern analysis; J.C.H. performed microscopy, transgenic plants generation and partially phenotyping; L.X.G. performed dsRNA cleavage activity assay, RNA immunoprecipitation sequencing and partially phenotyping; L.Q.P. and Y.Z. maintained the pathogens and plants and performed sample collection for sequencing; C.C.S. performed protein expression and purification; C.J.Z. and L.R. performed partially phenotyping; L.J.L. and X.Z. wrote the manuscript with improvement from S.Y.L., X.L.D. and X.P.F.

## Funding

This study was supported by the National Natural Science Foundation of China (32170366, 32270257), Biological Breeding–Major Projects (2023ZD04070), Agricultural Science and Technology Innovation Program of the Chinese Academy of Agricultural Sciences (CAAS‐CSNCB‐202303, CAAS‐ASTIP‐2021‐OCRI), Knowledge Innovation Special Project of Wuhan (2022020801010296, 2023020201020399), and China Agriculture Research System of MOF and MARA (CARS‐12).

## Conflicts of Interest

The authors declare no conflicts of interest.

## Supporting information




**Supporting File 1**: advs77476‐sup‐0001‐SuppMat.docx.

**Supporting File 2**: advs77476‐sup‐0002‐TablesS1‐S13.xlsx.

## Data Availability

The data that support the findings of this study are openly available in NCBI Sequence Read Archive (SRA) at https://www.ncbi.nlm.nih.gov/sra, reference number 1080080.
