# Supplementary material for: RNAi in the Rhizarian Phytopathogen Plasmodiophora brassicae: The Causal Agent of Clubroot Disease in Cruciferous Crops
Source: Adv Sci (Weinh). 2026 Sep 6:e24028. Online ahead of print. doi: 10.1002/advs.202524028 (PMC13547706; doi:10.1002/advs.202524028)
Supplement: Supplementary file 1 [file ADVS-9999-e24028-s002.docx]

Supporting Information

**RNAi in the Rhizarian Phytopathogen *Plasmodiophora brassicae* -the Causal Agent of Clubroot Disease in Cruciferous Crops**

*Xiong Zhang, Yupo Wu, Jiachang Hou, Lixia Gao, Longqi Pan, Cuicui Shen, Chuanji Zhao, Feng Gao, Yi Zhang, Li Ren, Xiaoping Fang, Xiaoling Dun, Shengyi Liu, Lijiang Liu**

**Figure S1. Overview of the *PbJS* genome assembly.**

**A,** *K*-mer depth distribution and the heterozygosity analysis of the *PbJS* genome (K = 21). **B,** Comparison of the genome assemblies of the *Pb* isolates *PbJS* generated in this study and the previously published isolate *Pbe3*.^[66]^ **C,** Genome collinearity analysis between *PbJS* and *Pbe3*. Gray lines indicate synteny blocks between the two genomes.

**Figure S2. Pipeline for identifying *Pb* sRNA species in this study.**

Raw Illumina reads were processed using fastp v0.12.6 for adapter removal and low-quality base trimming. After quality control, the clean reads were aligned to the Arabidopsis genome and Rfam v. 14.0 database using Bowtie v1.0 to filter out host-derived reads and non-coding RNAs. The remaining reads were aligned to the *PbJS* reference genome and high-confidence mapped reads were considered *Pb* sRNAs. The identified *Pb* sRNAs were further classified into miRNAs, phasiRNA-like siRNAs, and hc-siRNA-like siRNAs using the known miRNA databases miRBase v21.0 and the software ShortStack v 3.8.2 and sRNAminer.

**Figure S3. Excluding the possibility that *Pb* sRNAs are degradation products of *Pb* transcripts.**

**A,** The number of expressed *Pb* genes at four representative life stages detected using transcriptome and sRNAome sequencing-derived reads, respectively. **B,** Venn diagram and correlation analysis of the expression levels of *Pb* genes detected by both transcriptome and sRNAome sequencing-derived reads. Gene expression levels are shown as log_2_FPKM values for transcriptome sequencing and log_2_RPM values for sRNAome sequencing. **C,** The number of expressed Arabidopsis genes at four representative life stages determined by transcriptome and sRNAome sequencing-derived reads, respectively. **D,** Venn diagram and correlation analysis of the expression levels of Arabidopsis genes detected by both transcriptome and sRNAome sequencing-derived reads.

**Figure S4. Molecular characteristics of nonredundant *Pb* sRNA species.**

**A,** Size distribution of nonredundant *Pb* sRNA clean reads identified from five *Pb* life stages. **B,** 5′-terminal nucleotide distributions of nonredundant sRNA species. **C,** Percentage of nonredundant sRNA-producing loci in the *PbJS* genome at five life stages. Inter, intergenic regions; TE, transposon elements.

**Figure S5. Multiple sequence alignment of PbAGO1, PbAGO2, and representative AGO proteins.**

Multiple sequence alignment of the PIWI domains of PbAGO1, PbAGO2, and representative AGO proteins was generated using MUSCLE and visualized in Jalview. The conserved Asp-Asp-His (DDH) residues characteristic of slicer-active AGO proteins are highlighted in red.

**Figure S6. Design of RNAi target sequences for *PbAGO1*, *PbAGO2*, *PbDRL*, *PbCHS3*, and *PbCHS7*.**

**A,** Sense and antisense fragments of the targeted genes were respectively inserted into the HIGS vector containing a hairpin dsRNA-producing cassette. The position and sequence length of the fragmented sequence for gene silencing are indicated by a purple rectangular box in each targeted gene model. **B,** The CDS regions of *PbCHS3* and *PbCHS7* were aligned and the fragments sharing the highest sequence similarity (79.4% identity, highlighted with the red box) were selected as the RNAi target sequence to simultaneously silence both *PbCHS3* and *PbCHS7*. Pairwise alignment of *PbCHS3* and *PbCHS7* was generated using Muscle and visualized in JalView. Conserved nucleotides are highlighted in blue.

**Figure S7. Validation of target-specific dsRNA-mediated silencing of *PbAGO1* and *PbAGO2*.**

A-B, Validation of dsRNA-mediated silencing specificity in *N. benthamiana* leaves transiently expressing *PbAGO1* or *PbAGO2*. **C-D**, Effects of exogenous dsRNA-mediated silencing of *PbAGO1* or *PbAGO2* on *Pb* pathogenicity. *Pb*-infected Arabidopsis plants were harvested at 20 dpi for root phenotype observation and disease index analysis. Scale bars in C = 1 cm. **E**, Effects of *PbAGO1* or *PbAGO2* silencing on the accumulation of *Pb* sRNAs, as revealed by stem-loop RT-qPCR. **F**, Effects of *PbAGO1* or *PbAGO2* silencing on the expression levels of sRNA-regulated target genes, as revealed by RT-qPCR. For **A**, **B**, **E**, and **F**, data are presented as mean ± SD from three biological replicates, and statistical significance was determined by a two-sided Student’s *t*-test. For **D**, data are presented as mean ± SD from three biological replicates, each including 30 plants, and statistical significance was determined by a two-sided Student’s *t*-test. **P* < 0.05, ***P* < 0.01.

**Figure S8. Western blot validation of tagged PbAGO1, PbAGO2, and PbDRL protein expression.**

**A**, Western blot analysis of FLAG-tagged PbAGO1 and PbAGO2 proteins expressed in Expi293F cells. PbAGO1-FLAG and PbAGO2-FLAG indicate C-terminally FLAG-tagged proteins, whereas FLAG-PbAGO1 and FLAG-PbAGO2 indicate N-terminally FLAG-tagged proteins. Protein expression was detected using an anti-FLAG antibody. **B**, Western blot analysis of PbDRL carrying an N-terminal Strep tag and a C-terminal His10 tag expressed in insect cells. PbDRL expression was detected using an anti-Strep antibody.

**Figure S9. Analysis of PbAGO1- and PbAGO2-associated sRNAs by AGO immunoprecipitation sequencing.**

**A**, Correlation analysis of PbAGO1- and PbAGO2-associated sRNAs based on normalized read abundance. **B**, Length distribution of PbAGO1- and PbAGO2-associated sRNA reads. **C**, 5′-terminal nucleotide bias analysis of PbAGO1- and PbAGO2-associated sRNA reads.

**Figure S10. Phylogenetic analysis of RNase III-containing proteins from *Pb* and representative species.**

**A,** A maximum-likelihood phylogenetic tree was constructed using the alignment of 17 full-length RNase III-containing proteins from *Pb* and representative species. The tree was constructed by MEGA X with the JTT model. Bootstrap values >50% are shown at nodes. **B,** A structure tree was constructed based on the 3D structural similarity of all selected RNase III-containing proteins. The structure tree was generated using the DALI server.

**Figure S11. Phylogenetic analysis of the RNase III domains of PbDRL, Rnt1, Drosha, and Dicer proteins from representative species.**

**A,** A maximum-likelihood phylogenetic tree was constructed based on the sequence alignment of RNase III domains of selected proteins. The tree was generated by MEGA X with the JTT model. Bootstrap values >50% are shown at nodes. Subclades are distinguished by different background colors: rose red, RNase IIIa domain; ice blue, RNase IIIb domain; light green, RNase III domain of budding yeast Rnt1. The scale bar indicates the evolutionary distance in amino acid substitutions per site. **B,** Identification of the “Bump” helix motif of Drosha proteins by sequence alignment. Amino acids highlighted in red indicate the “Bump” helix motif inserted in the RNase IIIa domain of Drosha proteins but not in that of the Dicer proteins. Multiple sequence alignment of four representative Drosha and 11 Dicer proteins was generated using Muscle and visualized in JalView. Conserved residues are highlighted in blue.

**Figure S12. Molecular characterization of dsRBD-containing proteins in the *Pb* genome.**

**A,** Domain architectures of two dsRBD-containing proteins, Pb09092 and Pb07475, in *Pb*. **B,** Validation of the interaction between PbDRL and Pb09092 or Pb07475 by yeast two-hybrid assay.

**Table S1. Overview of the sequencing data for the *PbJS* genome assembly.**

**Table S2. Overview of the sequencing data for transcriptome analysis.**

**Table S3. Overview of the sequencing data for sRNAome analysis.**

**Table S4. Comparison of genome assemblies among four published *Pb* isolates.**

**Table S5. Genome-wide identification of the repetitive elements in the *PbJS* genome.**

**Table S6. Annotated phasiRNA-like loci in the *PbJS* genome.**

**Table S7.** **Annotated hc-siRNA-like loci in the *PbJS* genome.**

**Table S8. Annotated miRNAs and their genomic loci in the PbJS genome.**

**Table S9. Prediction of the miRNA target genes in the *PbJS* genome.**

**Table S10. Primer sequences used in this study.**

**Table S11. Probe sequences for northern blot analysis of sRNAs in this study.**

**Table S12. Protein information for identification of the core RNAi pathway proteins in the *PbJS* genome.**

**Table S13. Protein information for identification of chitin synthase genes in the *PbJS* genome.**
